# Report of the Commissioners of the Lunatic Asylum, or Indiana Hospital for the Insane, to the General Assembly

**Published:** 1846-01

**Authors:** 


### BIBLIOGRAPHICAL NOTICES.

*Report of the Commissioners of the Lunatic Asylum, or Indiana Hospital for the Insane, to the General Assembly. December,* 1845. (From Dr. Evans.)

This is a Report of a Board of Commissioners, consisting of John Evans, M. D., L. Dunlap, M. D., and James Blake, Esq., appointed at the last meeting of the General Assembly of Indiana, to purchase a site for a State Lunatic Asylum, and report a plan for a building, its probable cost, &c. &c. To aid in their task, a tour was made during the last summer by Dr. Evans, with a view to collect the practical information required, by visiting the best conducted Asylums for the insane in the United States, and advising with those learned in that department of medical science. The Report contains a synopsis of the results of this tour, and in small space presents the best devised plans for ventilation, cleanliness, watering, and surveillance.

The Report also informs us that a site has been chosen and purchased, combining all required advantages of healthfulness, locality, and beauty of scenery, drainage, and richness of soil. It is situated two miles west of Indianapolis, on the McAdamized National Road. A plan for the building was submitted with the Report, proposals have been received for the supply of material, and the amount raised by taxation is supposed to be sufficient for the active prosecution of the work during the ensuing season. We presume from this that Indiana will soon be able to boast of an asylum for the insane adequate to all the demands which will be made upon her philanthrophy for many years. May we hope that other Western States, our own included, will follow in her footsteps, and imitate her good example? ED.

